# The Multifocal On- and Off-Responses in the Human Diabetic Retina

**DOI:** 10.1371/journal.pone.0155071

**Published:** 2016-05-17

**Authors:** Jenny C. Y. Lung, Peter G. Swann, Henry H. L. Chan

**Affiliations:** 1 Laboratory of Experimental Optometry (Neuroscience), School of Optometry, The Hong Kong Polytechnic University, Hong Kong SAR, China; 2 School of Optometry, The Queensland University of Technology, Queensland, Australia; University of Houston, UNITED STATES

## Abstract

The characteristics of the on- and off-responses in the human diabetic retina by a “long-duration” multifocal electroretinogram (mfERG) paradigm were investigated. Changes in the retinal antagonistic interaction were also evaluated in the early stage of diabetes mellitus (DM). Twenty type II diabetic patients with no or mild non-proliferative diabetic retinopathy (NPDR) and twenty-one age-matched healthy controls were recruited for “long-duration” mfERG measurements. A 61-hexagon mfERG stimulus was displayed under two chromatic conditions (white/black and blue/black) at matched luminance. The amplitudes and implicit times of the on-response components (N1, P1 and N2) and off-response (P2) components were analysed. The blue stimulation generally triggered greater mfERG amplitudes in P1, N2 and P2 (p<0.05) than those from white stimulation in both control and diabetic groups. The diabetic group showed significantly greater N2 amplitude than the controls under white stimulation in mid-retinal regions (Rings 2 and 4) (p<0.05). When the stimulus was changed from white to blue, the diabetic group showed a smaller percentage change in N2 amplitude than the controls in peripheral retinal region (Ring 5) (p<0.02). When a stimulus is changed from white (broad-band spectral stimulation) to blue (narrow-band spectral stimulation), a decrease in the involvement of lateral antagonism would be expected. The larger amplitude of the on-response component (N2) in the diabetic patients suggested an imbalance of lateral antagonism, and the lesser percentage change of N2 amplitude in the diabetic group may indicate an impairment of the cross-talk at the middle retinal level in early stages of DM.

## Introduction

Diabetic retinopathy (DR) is an ocular vascular complication associated with diabetes mellitus (DM) which has a rapid increase of incidence in Asia [1]. DR is one of the leading causes of blindness among the working population [2]. It is proposed that damage of the vascular endothelium and pericytes, as well as the acceleration of cellular apoptosis, contribute to vascular leakage in the retina and hence the clinically visible signs of DR [3].

Early functional and structural deficits in the eye have been found in diabetic patients. Colour discrimination ability was shown to deteriorate with or without DR [4–6]. A generalized loss of chromatic discrimination in diabetic patients was reported [7], with a more pronounced reduction in the short-wavelength colour [4,8–9]. Indeed, selective loss of short-wavelength cone sensitivity was illustrated by electrophysiological assessments in diabetic patients [8–9]. Besides colour vision impairment, reduction of sensitivity in automated perimetry [10–11] and thinning of the retinal nerve fiber layer (RNFL) [12–16] were also reported.

The multifocal electroretinogram (mfERG), an objective assessment of retinal function with topographic details [17], was found to be superior to two standard clinical assessments (automated white-on-white perimetry and RNFL thickness measurement by optical coherence tomography) in demonstrating early physiological changes prior to any visible vascular lesions [18–22]. The mfERG reflects neural activities mainly in the middle and inner retinal layers [23]. Functional deterioration detected by the mfERG indicates disturbance of retinal adaptation at or beyond the level of secondary neurons. A number of studies have applied the mfERG with different protocols to provide objective evaluation of the functional changes in diabetic patients [18–20,24–35]. In fact, retinal dysfunction detected by the mfERG in diabetic patients was not fully explained by either weaker perimetry luminance sensitivity or morphological changes of the RNFL [22,29]. Thus, it is believed that the possible site for the functional changes is within the middle to inner layers of the retina.

To investigate the functional changes in the middle and inner retinal layers using mfERG, segregation of the on- and off-pathway responses is necessary. However, there is usually a large overlap of the two responses in the conventional mfERG paradigm. In order to dissociate the on- and off-pathways, Kondo and Miyake [36] modified the mfERG paradigm to mimic the “long-duration” flash which is applied in the Ganzfeld full-field electroretinogram [37]. By increasing the number of multifocal flashes and dark frames, the overlap between the on-response (the retinal signal measured when the light stimulus turns on) and off-response (the retinal signal measured when the light stimulus turns off) in the mfERG can be minimized. By this way, mfERG can be used to assess the on- and off-pathways separately [36]. Such a protocol has been successfully applied in some other retinal disorders to study the activities of the on- and off-pathways [38–40].

Different chromatic stimulations were studied in the electroretinogram measurements and it was demonstrated that narrow-band spectral stimulation can trigger a larger retinal signal than broad-band stimulation. It was suggested that the chromatic stimulations further isolated the antagonistic interactions initiated by networks of long-, middle- and short-wavelength cones and their secondary neurons [41].

In the present study, the modified long-duration mfERG paradigm described above was used to study the on- and off-responses of the middle and inner retinal layers in diabetic patients. Furthermore, the mfERG stimulus pattern was used under two chromatic conditions (white/black and blue/black) to manipulate any antagonistic interactions, in order to evaluate the changes of the cross-talk at or beyond the middle retinal level in the early stages of DM.

## Methods

### Subjects

Twenty type II diabetic patients (aged 46.6±7.4 years) with no or mild non-proliferative diabetic retinopathy (NPDR) and twenty-one age-matched healthy controls (aged 46.6±7.4 years) were recruited for this study. All of the participants had visual acuity equal to or better than 6/9 and their refractive errors were between +3.00D and -6.00D with astigmatism less than -1.25D. The mean DM duration of the diabetic patients was about 5.0±5.2 years (ranged from 1 to 21 years) based on patients’ reported history. All of them were under oral medication without insulin treatment. A thorough eye examination (including dilated fundus examination and biomicroscopy) was carried out for each subject. Fundus photo-documentation was performed in the central retina and in the eight cardinal gaze directions using a fundus camera (Topcon TRC-NW6S Non-Mydriatic Fundus Camera with software IMAGEnet, Japan). Each photo covered 45-degree field of view and totally nine non-stereoscopic fundus photos were taken for each eye. The fundus photos were then graded by a masked retinal specialist (PS) according to the ETDRS levels for classifying the presence of DR [42–43]. Participants with ocular diseases other than DR were excluded. Participants with long-term medication (including hypertension) other than for DM were excluded.

All of the study procedures fulfilled the tenets of the Declaration of Hesinki. This study was approved by the Human Ethics Committee of The Hong Kong Polytechnic University. Written consent was obtained from each individual subject after full explanation of the experimental procedures.

### Measurements

#### 1) Multifocal ERG recording

**Fig 1:**
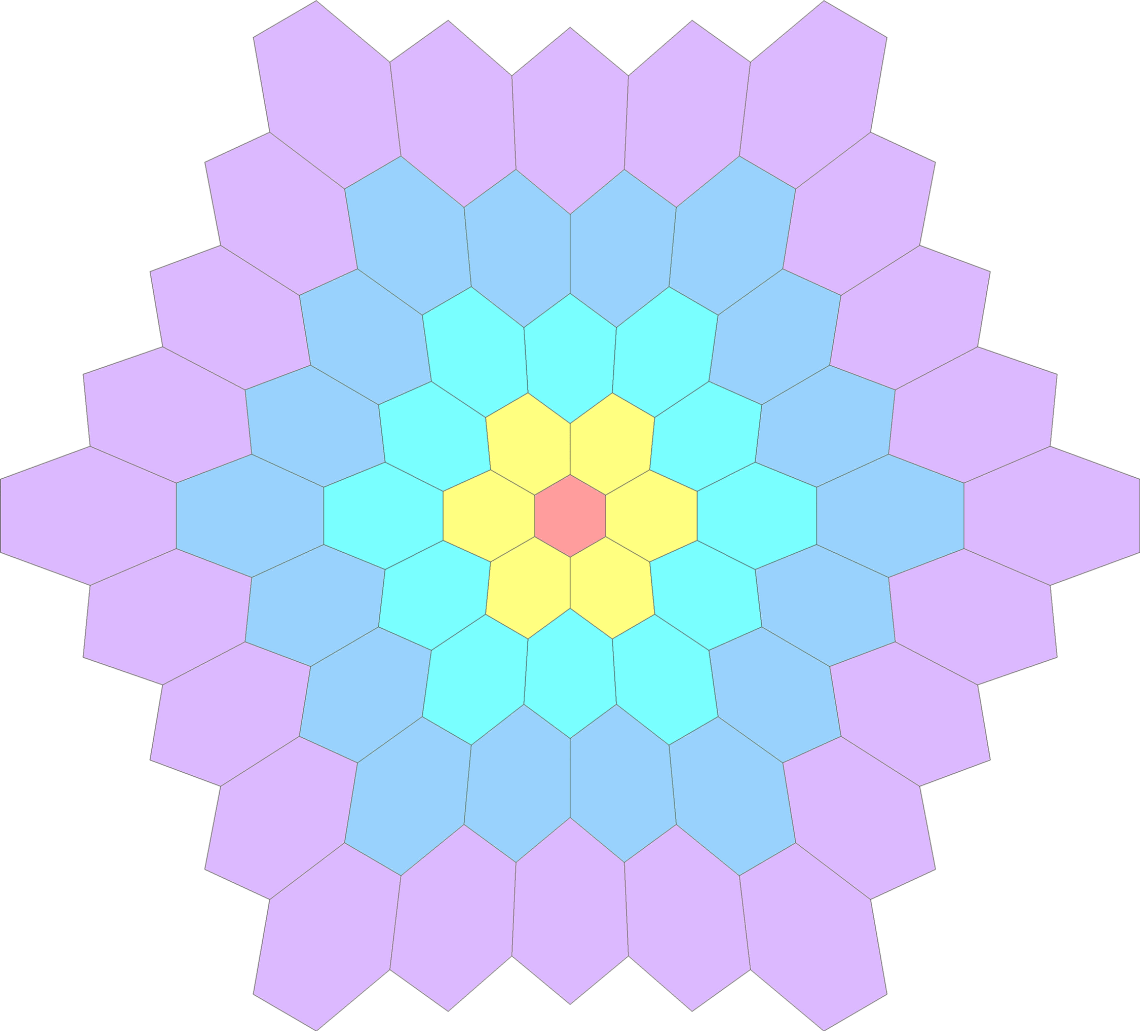
The 61-hexagonal mfERG stimulus pattern which was divided into five rings for data analysis.

The VERIS Science 5.1 system (Electro- Diagnostic- Imaging, Redwood City, CA, USA) was used for the “long-duration” mfERG measurement. A scaled 61-hexagon pattern (Fig 1) which subtended 49° horizontally and 47° vertically was displayed on a high luminance CRT monitor with P45 phosphor (FIMI Medical Electrical Equipment, Saronno, Italy). Each base period of the mfERG stimulation contained 16 video frames: 8 successive multifocal flash frames and 8 successive dark frames, as suggested by Kondo and Miyake [36,38]. Each base period lasted about 213.3 ms (bright phase duration: 106.6 ms, dark phase duration: 106.6 ms). The stimulus was displayed at a frame rate of 75 Hz and the pseudo-random m-sequence chosen had 2^11^−1 steps. The mfERG signal was amplified by 100,000X (Grass Instrument Co., Quincy, MA, USA) and bandpass filtered from 3 to 300 Hz with spatial averaging (iteration = 1) done by VERIS system which has monthly regular calibration.

Measurements were made under two chromatic conditions (white and blue stimulations). In the white condition, the bright phase was displayed at 22 cd/m^2^ and the dark phase was displayed at 0.3 cd/m^2^. The background luminance was maintained at the mean luminance level at 10 cd/m^2^. The room illuminance was dimmed and maintained at about 10 lux (eye level of the subject during mfERG recording).

In order to achieve the blue mfERG stimulation, the CRT monitor brightness was firstly turned up with the white hexagon at about 380 cd/m^2^ and the black hexagon at about 3.4 cd/m^2^. A Wratten 47A filter was inserted between the display and the eye to achieve the blue stimulation conditions. The Wratten 47A filter has peak transmission at 440nm, with cutoff at half-height at about 500nm [44]. The spectrum of visible wavelengths emitted from P45 phosphor (Y_2_O_2_S: Tb) [45] is broader than the cutoff of the Wratten 47A filter, and the combination of P45 and Wratten 47A produces a deep blue stimulus which stimulates mainly the short-wavelength (SW) and partly middle-wavelength (MW) cone systems [45–46]. The luminance levels of the white and black hexagons on the FIMI monitor under the 47A filter were adjusted so that the similar luminance was achieved as for the white stimulation condition (without 47A filter). Hence, under the condition with 47A filter, the resultant luminance of the white hexagon was about 21 cd/m^2^ while the black hexagon was about 0.3 cd/m^2^ with the background luminance of about 10 cd/m^2^. The luminance measurements were made using a Konica Minolta LS-110 photometer (Konica Minolta Optics, Inc., Tokyo, Japan). Subjects completed two mfERG recording sessions, one using the white stimuli and one using the blue stimuli. The order of presentation, whether using the white stimuli or the blue stimuli in the first recording session, was randomized.

One eye of each subject was randomly selected for mfERG measurement and dilated using 1% Tropicamide (Alcon, Fort Worth, TX, U.S.A.) until the pupil reached at least 7 mm diameter. A Dawson-Trick-Litzkow (DTL) electrode was used as the active electrode. A gold-cup electrode was placed at the outer canthus as the reference electrode, and another at the central forehead as the ground electrode. Subjects were corrected for the viewing distance of 33 cm using ophthalmic lenses. Each subject was instructed to fixate at the central cross of the mfERG stimulus pattern throughout the measurement. The measurement for each condition was divided into 32 segments. Any recorded segment contaminated by blinks or other artifacts was rejected and re-measured immediately. The central peak and the blindspot depression on the mfERG 3-D plot were used as an indicator of the signal quality based on the method described in a previous study [47].

#### 2) Measurement of the instantaneous plasma glucose level

The instantaneous plasma glucose level of the subjects was measured at the time of mfERG measurement. A user-friendly blood glucose meter (Accu-Chek Compact Plus, F. Hoffmann-La Roche Ltd, Basel, Switzerland) was applied on the finger tip to measure the plasma glucose level for each diabetic subject at least 2 hours after any food intake.

### Data analysis

**Fig 2:**
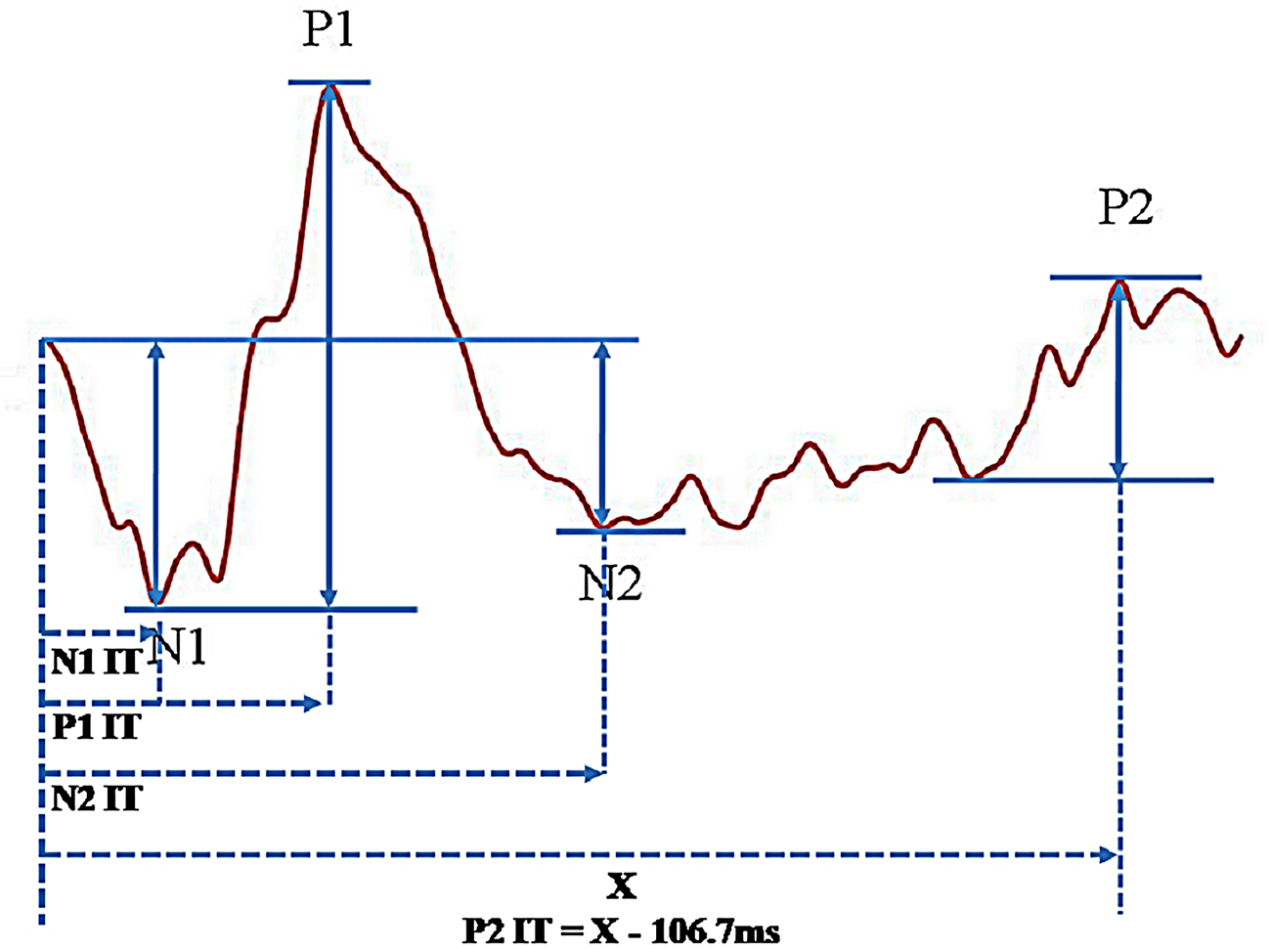
The waveform under the “long-duration” mfERG paradigm showing the parameters measured.

The mfERG responses from the 61-hexagon pattern were grouped into 5 rings for analysis. There were two sections of the resultant waveform: the on-response triggered by the mfERG bright phase (stimulus-on) and the off-response triggered by the mfERG dark phase (stimulus-off) (Fig 2). The on-response was the initial part of the waveform, which was similar to the conventional mfERG waveform, including a negative trough (N1) followed by a positive peak (P1) and then another trough (N2). After a plateau following N2, there was a second peak (P2) which was the off-response. The on-response (N1, P1 and N2) and off-response (P2) were defined according to the study by Kondo et al. [36]. The relevant amplitude and time parameters are shown in Fig 2. When the mfERG stimulus was changed from white to blue stimulation, the percentage changes for the mfERG responses were calculated using the following equation:

$$\frac{\left(\text{mfERG response under blue stimulus}\;–\;\text{mfERG response under white stimulus}\right)\;\text{x}\;100\%}{\left(\text{mfERG response under white stimulus}\right)}$$

This value was used to reflect the changes of the antagonistic interaction within the retina.

The statistical analysis was carried out using the software SPSS 16.0 (SPSS, Chicago, IL). Three factors were evaluated in this study. The within-subject factors were “Rings (5 rings)” and “Colours (white and blue)” while the between-subject factor was “Groups (control and diabetic groups)”. Three-way mixed design analysis of variance (ANOVA) was used for statistical analysis. Mauchly’s Test showed that the assumption of sphericity was violated, and conservative Greenhouse-Geisser correction, therefore, was applied. Post-hoc test with Bonferroni’s adjustment was used to account for the multiple comparisons. Statistical significant level of the adjusted p-value was set at < 0.05.

If the within-subject factors or their combinations showed significant effect(s) on the mfERG parameters, two-way repeated measures ANOVA (Factors “Colours” and “Rings”) were carried out to investigate which mfERG parameters were affected by the within-subject factors in each subject group. A further one-way ANOVA or unpaired t-test was carried out to locate the effect.

If the between-subject factor or its combination showed significant effect on the mfERG parameters, a mixed design two-way ANOVA (Factors “Rings” and “Groups”) was carried out to examine which mfERG parameters were affected in each colour stimulation. A further unpaired t-test was carried out to locate the significance level between subject groups.

For the percentage change of the mfERG responses, two-way mixed design ANOVA (Factors “Rings” and “Groups”) were carried out in order to compare the difference between two subject groups. When there was a significant group difference, further unpaired t-tests were carried out to find out its location(s).

## Results

Among the diabetic subjects, whose fundus photos were graded by the masked retinal specialist, 6 out of 20 were classified as “mild NPDR”, 2 as “no DR”, and 12 as between “mild NPDR” and “no DR” because only trace defects (less than 10 defects per subject) was found on the fundus photos and could not be defined as tiny microaneurysms. The averaged instantaneous plasma glucose level of the diabetic patients at the time of mfERG measurement was 8.7 ± 4.0 mmol/L (ranged from 5 to 19.2 mmol/L). As 14 out of 20 diabetic subjects had the conditions below mild NPDR, it was hard to further subdivide into smaller local groups to compare the mfERG results with the local fundus vascular changes. Hence, ring analysis was performed to investigate the physiological changes at different eccentricities in the diabetic retina.

### Effect of retinal eccentricity

Both the on- and off-responses mfERG amplitudes (N1, P1, N2 and P2) decreased with the increasing retinal eccentricity (Ring number).

**Fig 3:**
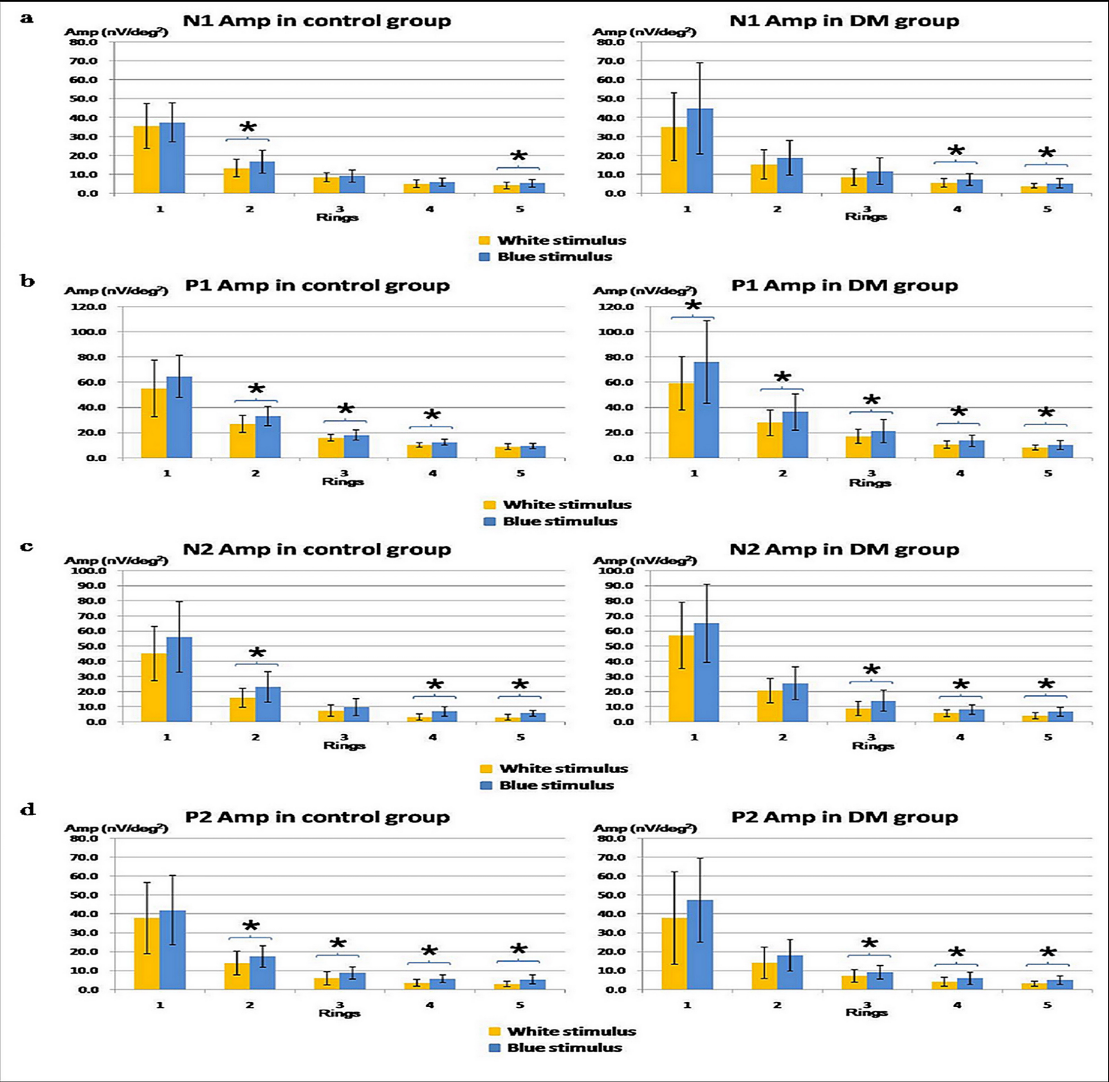
(a) The N1 amplitude (mean ± SD) in the DM and control groups under both the white and blue conditions; (b) The P1 amplitude (mean ± SD) in the DM and control groups under both the white and blue conditions; (c) The N2 amplitude (mean ± SD) in the DM and control groups under both the white and blue conditions; (d) The P2 amplitude (mean ± SD) in the DM and control groups under both the white and blue conditions (* indicates parameters that achieved statistically significant level with p < 0.05)

Three-way mixed design ANOVA (Colours x Rings x Groups) showed significant differences for all of the amplitudes (N1: F = 224.353, df = 1.151, p < 0.001; P1: F = 306.489, df = 1.161, p < 0.001; N2: F = 283.890, df = 1.185, p < 0.001; and P2: F = 183.391, df = 1.109, p < 0.001) with p < 0.05 (Fig 3A–3D). Further two-way repeated measures ANOVA (Colours x Rings) revealed a significant difference in both the control and diabetic groups. Post-hoc testing of ring amplitudes illustrated that the largest mfERG amplitudes for all measures was in Ring 1, the second largest in Ring 2 and the smallest in Ring 5 (p < 0.02) under both white and blue stimulation conditions. The third largest amplitudes under both stimulations were in Ring 3 (p < 0.01) in the control and diabetic groups except for the N2 amplitude under the blue stimulation in the control group.

In general, there was no obvious trend of the implicit times for the on- and off-response components across retinal eccentricity for either the control or diabetic groups. Three-way mixed design ANOVA showed that the factor “Rings” affected the on-response mfERG implicit time of N1 (F = 5.294, df = 2.730, p < 0.005), P1 (F = 35.248, df = 3.161, p < 0.001) and N2 (F = 9.026, df = 2.272, p < 0.001), while no significant difference was found in the implicit time of the off-response P2 (F = 0.673, df = 3.118, p > 0.5). Further two-way repeated measures ANOVA (Colours x Rings) and one-way repeated measures ANOVA (Rings) with Bonferroni’s post-hoc test indicated that the implicit time of P1 for the central Ring 1 was significantly longer than the peripheral Rings 3 and 4 (p < 0.01) in the control group under both stimulation conditions. In the diabetic group, the implicit times of P1 in central Rings 1 and 2 were found to be longer than in peripheral Rings 3 to 5 (p < 0.05) under blue stimulation only.

### Effect of chromatic (white and blue) mfERG stimulations

Three-way mixed design ANOVA indicated that the factor “Colours” had significant differential effects on the on- and off-response amplitudes with p < 0.01 (N1: F = 8.666, df = 1, p < 0.01; P1: F = 22.997, df = 1, p < 0.001; N2: F = 21.181, df = 1, p < 0.001; P2: F = 13.612, df = 1, p < 0.001).

In the control group, two-way repeated measures ANOVA (Colours x Rings) showed that the blue stimulation triggered larger P1 (p < 0.01), N2 (p < 0.05) and P2 (p < 0.05) amplitudes than the white stimulation. Further paired t-tests on the ring amplitudes were performed. For the P1 amplitude, a significant chromatic effect was found at Rings 2 to 4 (p < 0.05) (Fig 3B, left panel); for the N2 amplitude, a significant chromatic effect was found at Rings 2, 4 and 5 (p < 0.02) (Fig 3C, left panel); for the P2 amplitude, a significant chromatic effect was found at Rings 2 to 5 (p < 0.05) (Fig 3D, left panel).

In the diabetic group, two-way repeated measures ANOVA (Colours x Rings) showed that the blue stimulation triggered larger amplitudes for all mfERG responses than the white stimulation (p < 0.04). Further paired t-tests on the ring amplitudes revealed the location of the significant effect. For the N1 amplitude, there was a significant chromatic effect in Rings 4 to 5 (p < 0.01) (Fig 3A, right panel); for the P1 amplitude, this effect was found in all five rings (p < 0.02) (Fig 3B, right panel); for the N2 and P2 amplitudes, the chromatic effect was mainly found at Rings 3 to 5 (p < 0.01) (Fig 3C and 3D, right panels).

**Fig 4:**
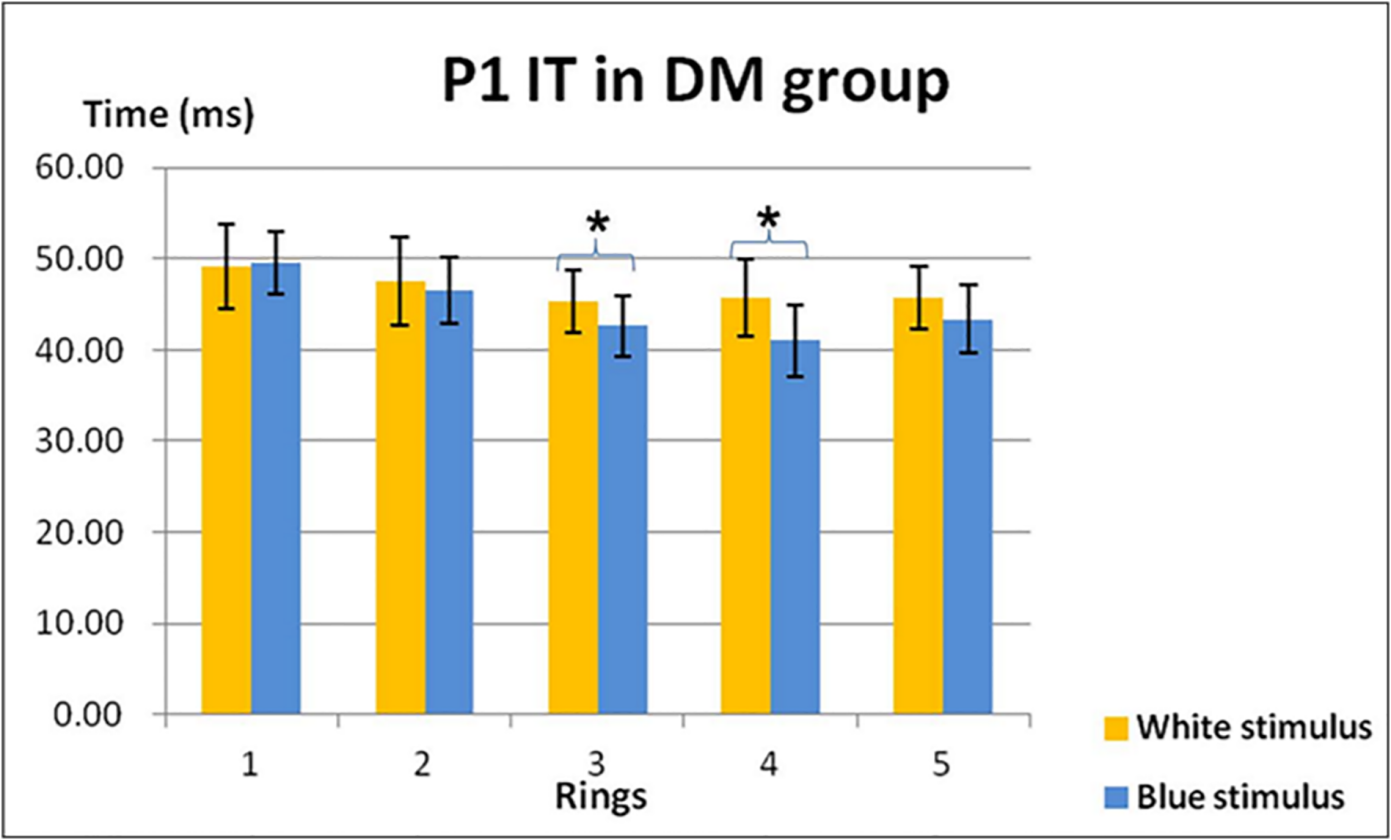
The P1 implicit time (mean ± SD) in the diabetic group under both the white and blue conditions (* indicates parameters that achieved statistically significant level with p < 0.05)

Under different chromatic stimuli, it was observed that the implicit times of the on-responses (P1 and N2) of the blue stimulation were shorter than those of the white stimulation in the diabetic group only. The chromatic factor had a statistically significant effect on the implicit time of P1 at Rings 3 and 4 only in the diabetic group. The P1 implicit time in response to the white stimulation was longer than the blue stimulation (p < 0.01) (Fig 4). No other consistent trend in implicit time was observed for the remaining mfERG components.

### Comparison between the control and diabetic groups

**Fig 5:**
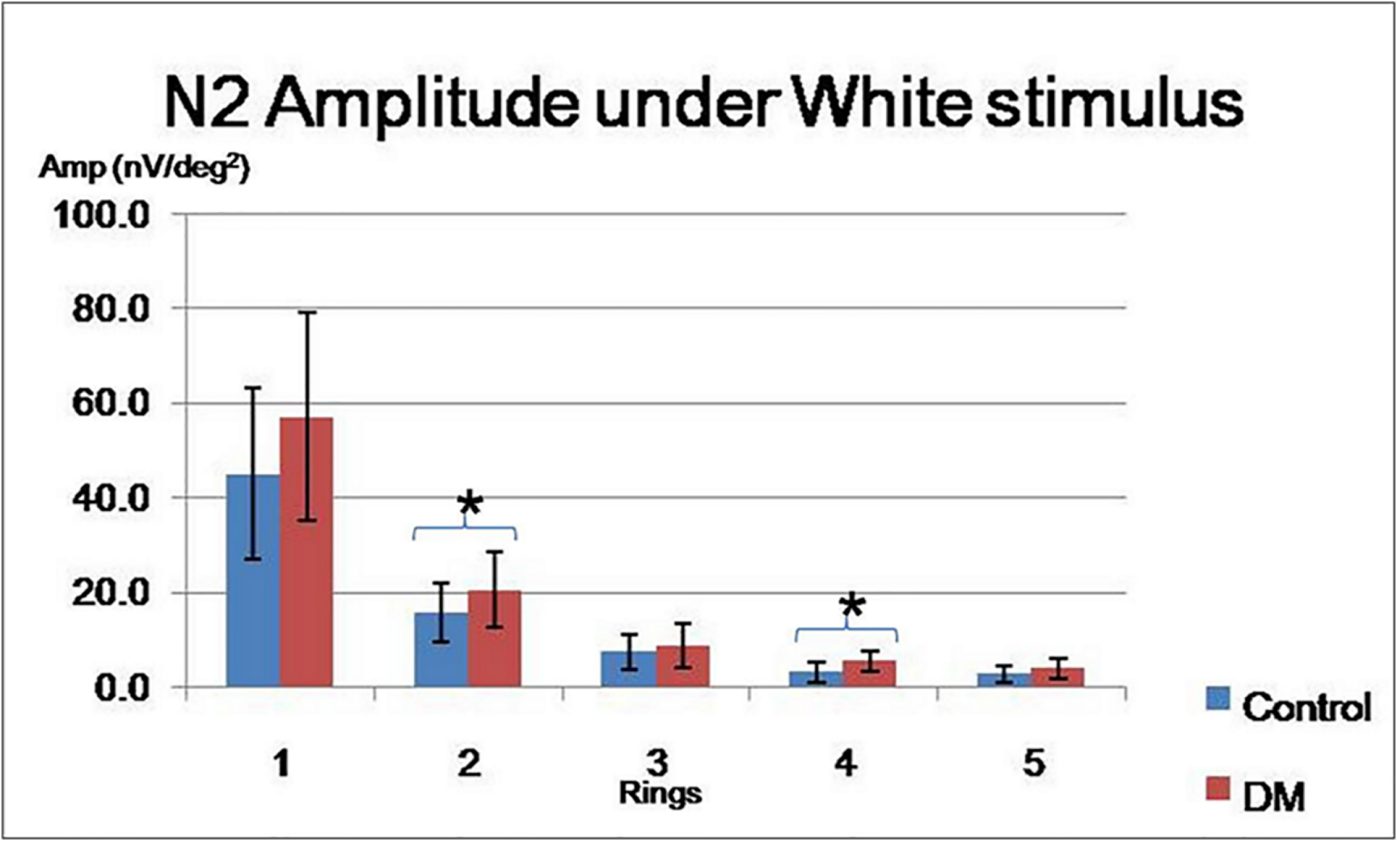
The N2 amplitude (mean ± SD) in the control and diabetic groups under the white condition (* indicates parameters that achieved statistically significant level with p < 0.05).

Three-way mixed design ANOVA showed no significant difference in the implicit time of the mfERG parameters (p > 0.20 with low observed power of 0.051), while the diabetic subjects showed significantly larger N2 amplitudes (F = 4.517, df = 1, p < 0.05) than did the healthy controls. Two-way mixed design ANOVA illustrated that there was a significant “Groups” difference only for white stimulation (p < 0.03). Unpaired t-tests showed that this effect achieved a statistical significant level at Ring 2 (p < 0.05) and Ring 4 (p < 0.001) (Fig 5).

**Fig 6:**
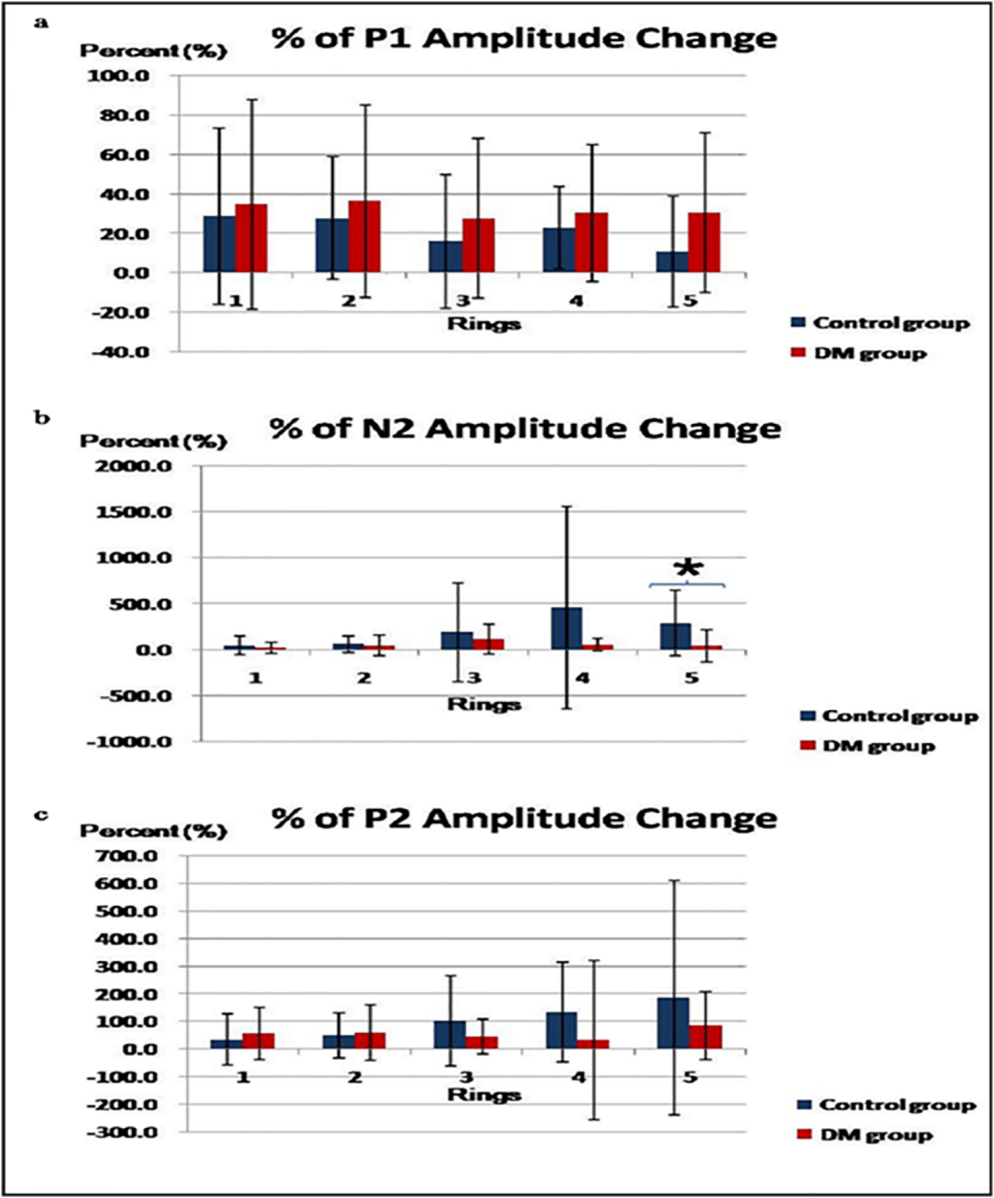
(a) The amplitude changes (in percentage) of the P1 amplitude (mean ± SD) due to the change from white to blue stimulus between the control and the diabetic groups; (b) The amplitude changes (in percentage) of the N2 amplitude (mean ± SD) due to the change from white to blue stimulus between the control and the diabetic groups; (c) The amplitude changes (in percentage) of the P2 amplitude (mean ± SD) due to the change from white to blue stimulus between the control and the diabetic groups (* indicates parameters that achieved statistically significant level with p < 0.05)

Considering the amplitude changes of major components (in percentage) when the stimulus was changed from white to blue, the P1 amplitude consistently showed a larger percentage increase across retinal eccentricity in the diabetic than in the control group (Fig 6A), while the on-response component N2 showed the opposite. Two-way mixed design ANOVA (Rings x Groups) revealed that the N2 amplitude of the diabetic group had a significantly smaller degree of increase than the control group (p < 0.02) especially for Ring 5 as demonstrated by the unpaired t-tests (Fig 6B).

For the P2 amplitude change, when the stimulation changed from white to blue, the diabetic group also had a greater increase than the control at Rings 1 and 2 but a lesser increase for Rings 3 to 5 as shown in Fig 6C; however, no statistical significance was shown in the two-way mixed design ANOVA (p > 0.05). No consistent trend was observed in the N1 amplitude change. Both the diabetic and control groups showed an increase in the P2 implicit time, but no consistent change was observed in the implicit time of the other mfERG components.

## Discussion

By using white and blue stimuli in the “long-duration” mfERG paradigm, waveforms containing two positive peaks were generated. These are similar to the resultant waveform in the long-duration full-field electroretinogram. This “long-duration” mfERG paradigm is based on the Ganzfeld full-field electroretinogram with a long-duration stimulus. The mfERG stimulus applied in this study was not a true continuous flash, but a series of flash impulses at high frequency. Saeki and Gouras [48] demonstrated that a high frequency flash series produces an effect similar to that produced by a light pulse of long-duration, which can generate a positive off-response.

In this study, the blue stimulus generally triggered a stronger mfERG signal in both diabetic and control groups. The mfERG paradigm was conducted at the same luminance level under white and blue conditions. The white stimulus has a broad-band spectrum which stimulates the long (L)-, middle (M)- and short (S)-wavelength sensitive cones; while the blue stimulus has a narrow-band spectrum and it mainly stimulates the SW sensitive cones. The Wratten 47A filter we used (λmax = 450um) minimized but did not totally eliminate the involvement of the L- and M-cone pathways. This would reduce the lateral antagonism between different cone pathways [41] and the reduction is expected to enhance the mfERG responses.

Due to the similarity of the “long-duration” protocols in the full-field electroretinogram and mfERG [36,41,48], it was proposed that the N2 trough found beyond the first P1 peak originates from the inner retinal layer, probably a region near the ganglion cell layer [41]. Under the “long-duration” mfERG paradigm, the overlap between the on- and off-pathway responses was minimized. However, the on- and off-responses were not completely dissociated. The on-response was predominantly from the depolarizing bipolar cells shaped by the hyperpolarizing bipolar cells, while the off-response was predominantly from the hyperpolarizing bipolar cells shaped by the depolarizing bipolar cells [36–37,41,49–50].

Under both the white and blue stimulation conditions, the N2 amplitude in the diabetic group was larger than that in the control group. The on-response component under blue stimulus conditions increased in the diabetic group. Four possibilities may lead to an increase of the N2 amplitude in the DM patients: 1) Stronger activity of the inner retina (near the ganglion cell layer); 2) Reduced on-pathway activity; 3) Enhanced off-pathway activity; 4) Weakening of the lateral antagonistic interaction. However, the first three points appear to be unable to explain the changes in the DM patients in our study. Previous morphological studies have shown that the inner retinal structure was altered in diabetic patients [13–16,51–56]. With increased neural apoptosis, a reduced rather than an increased inner retinal responses were reported [18–20,24–28,30–32,57]. Besides, the P1 and N1 amplitudes in the diabetic group were generally greater than the control group. This is counter to increase the on-pathway activity. Thus, the increased N2 amplitude cannot be simply explained solely by the changes in the activities of the ganglion cells, the on- or off-pathways. It is possible that the antagonistic interactions within retinal cellular components play a role in the enhancement of the N2 amplitude.

Considering the hypothesized changes in lateral antagonism, when the stimulus was changed from white (broad-band) to blue (narrow-band), it was proposed that there was a reduction in antagonistic interaction [41] and this resulted in an increase of the mfERG amplitude. This was reflected by the positive percentage amplitude change found in our results as seen in Fig 6A–6C.

The higher the percentage value of the mfERG amplitude change calculated in the results (Fig 6), the greater the reduction in lateral antagonistic interaction would be, due to the change of stimulus colours from white to blue. Comparing the percentage change of the mfERG amplitude in both subject groups, the control group initially showed a greater percentage change than the diabetic group in terms of the on-response P1, but a reverse trend was observed in the later on-response component N2 and the off-response component P2. It is suggested that both the components N2 and P2 may share the common origin, most likely the off-pathway, which exerts less antagonistic interaction to the on-pathway in the early stage of DM. These effects were consistent across the retina, but only reached significance at ring 5 (see Fig 6B). Whether the peripheral retina is more prone to this functional loss still needs further investigation. Moreover, although the trends of the changes were shown, the significant findings might depend on how the responses were grouped for analysis due to the relatively small sample size in our study.

Previous electroretinogram studies [18–20,24–34,57–63] reported a decrease in the inner retinal responses rather than an enhanced response. However, in this study, greater amplitude was recorded from the diabetic subjects when the blue stimulus was used. The dissociation of the on- and off-retinal pathway activities by the long-duration paradigm can allow detection of subtle changes in responses of the diabetic retina. It is believed that the changes or weakening of the inhibitory cross-talk between different retinal pathways will be reflected by this mfERG response paradigm. This may also explain why our findings did not show any obvious delay in the implicit time as compared to previous studies, which measured conventional mfERG response with the overlapping of the on- and off-responses. The relatively small sample size may also be an issue where a delay of response was not demonstrated. In the full-field electroretinogram, a decrease of photopic negative response has been reported for a brief blue flash stimulus [64]. The dark frames in this mfERG paradigm help eliminate the temporal adaptive effect of the successive flash stimuli, but there may be still a certain lateral effect from the neighboring hexagons which may affect the high-order retinal response. Thus the N2 component may not be directly comparable to the photopic negative response in the conventional full-field electroretinogram. Further studies on the long-duration full-field electroretinogram on diabetic animal models using pharmacological dissection of the response [23,37,41,50] should be performed in order to enhance our understanding of the cellular origin of the N2 component and to determine the possible surrogate biomarkers for the detection of early changes in DR.

## Conclusion

In this study, the “long-duration” mfERG paradigm showed that the on-response component N2 “increased” in the early stage of DM. By the use of broad- and narrow-band stimuli, it was further suggested that the weakened lateral antagonistic mechanism leads to an increase of the N2 amplitude. These response changes indicate that the middle retinal layers may be worthy of study in understanding the underlying pathological mechanism in the early stages of DM. This will also promote the development of novel therapies for DR in the future.

## Supporting Information

S1 Dataset (PDF)
