# Supplementary material for: The Multifocal On- and Off-Responses in the Human Diabetic Retina
Source: PLoS One. 2016 May 17;11(5):e0155071. doi: 10.1371/journal.pone.0155071 (PMC4871365; doi:10.1371/journal.pone.0155071)
Supplement: S1 Dataset — (PDF) [file pone.0155071.s001.pdf]

Gp	N1TW1	N1TW2	N1TW3	N1TW4	N1TW5	N1AW1	N1AW2	N1AW3	N1AW4	N1AW5	P1TW1	P1TW2	P1TW3	P1TW4	P1TW5	P1AW1	P1AW2	P1AW3
0	27.50	26.70	13.30	13.30	18.30	46.00	10.80	12.60	6.30	3.70	55.00	55.00	40.80	39.20	44.20	57.50	23.10	16.50
0	26.70	16.70	21.70	20.80	20.00	36.80	10.00	7.50	4.90	3.70	39.20	45.00	45.00	41.70	49.20	48.90	25.90	14.60
0	16.70	17.50	21.70	15.00	13.30	60.40	21.60	12.80	8.00	6.40	47.50	47.50	42.50	42.50	51.70	135.00	36.50	16.90
0	17.50	20.80	23.30	22.50	22.50	27.40	21.60	13.20	10.50	9.00	59.20	48.30	48.30	59.20	60.00	54.60	29.90	21.70
0	17.50	23.30	19.20	19.20	19.20	39.00	13.50	5.90	4.00	4.60	41.70	40.80	43.30	37.50	39.20	47.90	23.00	12.90
0	25.80	20.80	20.00	18.30	23.30	29.50	9.40	5.80	2.90	2.60	47.50	52.50	47.50	46.70	46.70	33.00	21.10	16.40
0	26.70	26.70	26.70	15.00	15.00	39.60	12.50	8.00	3.40	3.40	45.80	45.80	45.00	47.50	55.00	53.10	34.90	19.40
0	15.80	25.80	20.80	19.20	18.30	36.40	18.60	8.50	5.30	3.10	50.00	50.80	35.80	44.20	41.70	71.90	29.80	15.00
0	24.20	24.20	23.30	22.50	20.80	23.10	8.20	6.40	4.50	2.60	51.70	49.20	42.50	42.50	42.50	48.90	18.80	12.60
0	27.50	27.50	20.00	20.00	20.00	30.50	11.00	7.70	3.30	5.10	45.80	48.30	37.50	38.30	48.30	66.00	37.80	20.50
0	27.50	21.70	22.50	17.50	21.70	42.80	13.90	7.40	3.10	5.50	45.80	45.80	37.50	45.00	38.30	52.00	29.80	19.10
0	32.50	30.80	30.80	14.20	19.20	61.10	7.60	6.70	3.20	1.60	48.30	49.20	42.50	48.30	47.50	68.40	27.60	12.00
0	21.70	22.50	22.50	21.70	21.70	26.20	11.30	11.00	5.90	3.30	51.70	48.30	42.50	45.80	47.50	37.50	24.30	13.80
0	23.30	22.50	22.50	23.30	24.20	37.20	18.30	10.70	7.00	5.50	46.70	47.50	45.80	45.80	45.00	77.40	40.10	19.90
0	31.70	29.20	21.70	26.70	18.30	51.80	18.00	10.40	6.30	5.60	54.20	50.00	48.30	41.70	48.30	34.00	22.40	15.60
0	20.00	19.20	19.20	18.30	20.80	23.90	16.40	7.10	5.30	2.60	45.80	45.80	48.30	48.30	60.00	57.20	27.50	14.70
0	21.70	20.00	20.00	17.50	17.50	23.20	9.40	8.10	6.50	5.20	48.30	48.30	41.70	42.50	42.50	51.90	28.80	15.60
0	15.80	16.70	15.80	19.20	20.00	33.60	15.10	5.70	2.50	3.30	50.00	46.70	45.80	40.80	40.00	51.30	23.60	14.10
0	25.00	25.80	17.50	17.50	23.30	17.70	5.70	7.80	4.10	2.20	55.00	46.70	40.00	45.80	41.70	35.90	18.10	13.80
0	22.50	22.50	22.50	20.80	21.70	32.10	15.90	7.30	4.20	3.20	40.80	40.80	40.80	40.80	40.80	33.80	23.50	15.20
0	30.80	24.20	30.80	30.00	22.50	26.30	8.40	5.80	2.90	2.90	48.30	48.30	41.70	43.30	42.50	34.40	14.80	13.00
1	29.20	25.80	26.70	19.20	23.30	23.20	8.20	4.90	3.90	2.30	49.20	48.30	48.30	53.30	47.50	53.30	29.50	17.30
1	18.30	25.00	24.20	24.20	20.00	23.90	20.50	13.90	5.60	4.40	45.80	55.80	50.80	50.00	42.50	67.40	37.50	21.60
1	32.50	19.20	20.80	25.00	23.30	53.00	18.30	8.00	4.60	4.40	55.80	50.00	49.20	45.80	45.80	75.70	37.80	15.50
1	20.00	20.00	18.30	16.70	17.50	31.40	9.70	5.20	4.30	1.20	50.00	43.30	40.80	40.80	42.50	46.30	19.00	13.80
1	19.20	18.30	18.30	19.20	10.80	59.90	21.40	20.30	10.30	3.30	45.80	34.20	41.70	42.50	43.30	82.60	43.70	29.50
1	20.80	20.80	20.00	20.00	16.70	71.50	34.10	12.40	7.00	3.90	43.30	50.00	46.70	45.80	46.70	75.70	26.40	23.20
1	26.70	26.70	19.20	22.50	27.50	57.20	24.50	11.20	6.90	4.80	49.20	41.70	41.70	40.80	40.00	49.50	25.80	16.70
1	16.70	25.00	22.50	23.30	28.30	11.80	7.90	4.30	3.50	3.00	50.00	49.20	48.30	48.30	48.30	42.30	20.20	13.90
1	23.30	24.20	23.30	23.30	22.50	34.10	13.20	5.50	5.20	5.40	48.30	47.50	48.30	48.30	50.80	55.10	23.90	13.80
1	23.30	33.30	28.30	27.50	26.70	34.60	11.20	7.80	5.90	4.00	50.00	56.70	46.70	44.20	40.80	37.50	12.30	9.30
1	25.80	22.50	23.30	22.50	21.70	22.60	12.30	6.10	3.30	3.40	45.80	46.70	45.80	45.80	45.00	37.00	19.20	10.50
1	21.70	20.80	28.30	20.00	19.20	29.80	11.20	10.50	5.30	4.60	43.30	43.30	42.50	40.80	42.50	64.30	27.60	19.40
1	33.30	18.30	19.20	22.50	21.70	10.20	4.40	4.10	2.60	2.70	59.20	47.50	45.80	39.20	45.80	29.20	22.40	16.20
1	16.70	24.20	24.20	19.20	22.50	17.50	7.50	5.00	2.30	3.00	47.50	48.30	40.00	48.30	50.00	43.50	22.10	13.90

1	22.50	23.30	23.30	24.20	24.20	19.50	8.80	5.60	5.00	3.70	43.30	49.20	49.20	50.80	49.20	31.70	15.70	11.10
1	31.70	31.70	30.80	23.30	23.30	33.80	13.30	5.60	5.90	3.90	50.80	50.00	43.30	50.80	50.00	51.60	18.20	10.80
1	26.70	20.80	20.00	31.70	28.30	38.60	19.90	13.80	7.50	6.20	47.50	43.30	43.30	48.30	48.30	86.20	33.40	23.20
1	12.50	17.50	19.20	15.00	14.20	37.90	19.20	4.40	6.40	4.70	47.50	48.30	47.50	47.50	41.70	102.00	49.70	24.40
1	25.80	19.20	20.00	10.80	17.50	67.70	28.10	13.60	10.10	5.50	60.00	47.50	47.50	38.30	50.00	94.60	42.10	23.00
1	17.50	16.70	13.30	15.80	21.70	23.90	10.60	7.60	3.20	2.60	50.00	50.00	39.20	45.00	44.20	53.50	27.70	11.10

P1AW4	P1AW5	N2TW1	N2TW2	N2TW3	N2TW4	N2TW5	N2AW1	N2AW2	N2AW3	N2AW4	N2AW5	P2TW1	P2TW2	P2TW3	P2TW4	P2TW5	P2AW1
12.00	9.10	71.70	71.70	71.70	64.20	58.30	63.20	25.30	14.10	3.70	3.00	20.03	20.03	19.13	23.33	23.33	33.40
8.80	6.40	69.20	65.00	63.30	73.30	74.20	38.80	10.40	5.30	3.20	0.40	21.63	15.83	16.63	17.53	10.83	35.30
13.00	12.60	85.00	67.50	69.20	70.00	70.80	68.30	29.90	10.60	6.60	6.80	27.53	26.63	26.63	27.53	27.53	93.60
15.40	14.70	86.70	68.30	70.00	90.80	100.00	56.20	22.80	5.20	0.20	2.90	10.83	17.53	19.13	24.13	15.83	75.00
7.40	7.70	72.50	61.70	60.80	55.80	60.00	61.10	21.40	6.40	1.50	1.50	16.63	31.63	21.63	20.83	21.63	27.10
10.80	7.30	84.20	85.80	68.30	98.30	109.20	48.10	10.90	0.40	1.60	4.40	29.13	28.33	28.33	30.03	34.13	47.30
10.20	12.10	74.20	75.00	98.30	103.30	102.50	23.80	8.60	2.50	1.50	3.30	26.63	40.03	40.03	43.33	36.63	25.70
11.10	9.10	76.70	87.50	60.80	101.70	115.80	18.20	8.20	9.30	5.70	6.10	30.83	30.03	30.03	38.33	31.63	19.40
9.30	7.70	82.50	82.50	73.30	69.20	93.30	39.50	18.10	6.70	1.70	1.40	23.33	27.53	21.63	19.13	17.53	24.70
9.90	9.00	80.80	68.30	73.30	68.30	101.70	59.50	17.00	7.60	1.10	2.40	9.13	9.13	10.83	10.83	11.63	17.20
11.20	9.40	65.00	74.20	78.30	102.50	99.20	75.30	25.50	2.00	3.90	3.70	22.53	15.83	25.03	20.03	19.13	39.70
9.50	10.60	74.20	68.30	69.20	95.80	104.20	67.10	11.00	14.50	5.20	1.60	31.63	30.03	30.03	35.03	35.03	53.10
9.50	7.20	71.70	71.70	72.50	80.00	101.70	40.90	13.60	8.70	3.60	1.80	13.33	25.03	23.33	24.13	25.83	18.10
10.70	9.70	82.50	80.80	71.70	71.70	101.70	14.80	9.00	5.60	3.60	5.50	47.33	35.83	36.63	47.53	47.53	24.80
10.10	8.60	78.30	72.50	75.00	74.20	57.50	63.20	20.60	10.20	0.70	0.80	29.13	29.13	30.03	23.33	23.33	49.90
9.10	7.30	70.00	69.20	69.20	100.80	100.80	30.20	14.30	5.60	2.70	1.20	24.13	25.83	25.83	25.83	25.83	27.90
10.90	8.40	88.30	82.50	68.30	69.20	91.70	41.40	12.60	9.10	5.20	3.50	33.33	33.33	33.33	23.33	22.53	47.80
8.70	6.90	71.70	85.00	84.20	71.70	60.80	24.20	11.30	3.80	0.50	0.40	40.03	38.33	36.63	34.13	40.03	32.20
7.90	5.80	89.20	72.50	72.50	88.30	52.50	23.70	9.40	7.00	4.10	0.90	26.63	23.33	20.83	31.63	30.83	37.80
8.70	6.30	65.80	65.80	65.00	67.50	60.00	41.50	15.40	5.60	1.30	0.80	13.33	12.53	10.83	20.83	22.53	24.40
7.60	5.90	89.20	73.30	64.20	90.00	90.00	47.10	16.50	12.00	6.70	4.60	32.53	32.53	38.33	37.53	35.83	37.10
12.00	10.00	84.20	80.80	100.00	99.20	99.20	49.00	12.70	3.20	2.70	1.50	27.53	27.53	26.63	35.83	35.83	14.70
12.20	8.10	84.20	80.00	67.50	68.30	97.50	58.80	26.30	11.50	5.20	4.50	27.53	16.63	15.83	15.03	15.03	35.50
8.80	10.20	70.80	69.20	66.70	92.50	97.50	46.90	6.30	6.90	5.90	5.20	15.03	15.03	14.13	15.03	17.53	26.30
12.40	7.40	74.20	78.30	89.20	89.20	93.30	54.20	16.50	7.30	6.60	3.60	23.33	24.13	21.63	22.53	23.33	10.10
14.20	9.80	94.20	85.00	64.20	81.70	85.00	68.90	25.20	18.70	8.70	3.70	34.13	34.13	35.03	39.13	39.13	83.20
15.20	10.10	81.70	71.70	70.00	70.00	70.80	87.00	36.70	17.90	9.10	4.60	15.03	17.53	18.33	20.03	20.03	40.70
11.10	7.40	71.70	66.70	65.80	67.50	67.50	77.70	33.80	10.90	9.10	6.60	22.53	22.53	23.33	19.13	20.03	64.40
8.40	5.30	70.80	70.80	70.00	99.20	100.00	30.40	13.10	1.80	2.50	2.90	25.83	26.63	24.13	28.33	26.63	31.30
9.50	10.50	82.50	80.80	75.80	70.00	68.30	60.00	18.10	5.00	5.10	1.70	40.83	34.13	34.13	34.13	25.83	42.20
4.90	3.50	79.20	83.30	86.70	69.20	55.80	60.50	20.80	10.30	5.20	3.00	30.03	27.53	25.83	21.63	23.33	28.30
7.10	6.60	81.70	75.80	66.70	66.70	80.80	34.20	12.20	5.60	4.10	3.50	15.83	15.83	14.13	15.83	16.63	14.90
12.60	10.10	86.70	85.80	62.50	56.70	100.00	68.00	30.90	13.70	5.00	6.10	20.83	20.83	22.53	18.33	17.53	30.50
9.30	7.50	76.70	75.80	69.20	63.30	63.30	56.40	15.80	4.50	3.00	-0.80	27.53	29.13	20.83	20.03	25.83	13.80
8.50	6.90	80.80	80.00	76.70	100.80	101.70	58.20	23.00	6.90	3.00	3.00	20.03	20.83	22.53	23.33	25.03	25.60

8.20	7.90	80.00	80.80	105.00	103.30	105.00	33.80	13.20	2.40	2.30	3.40	29.13	29.13	24.13	23.33	22.53	19.50
8.10	7.10	59.20	73.30	97.50	70.00	69.20	30.30	18.50	10.60	6.60	4.00	43.33	42.53	42.53	43.33	49.13	43.30
13.70	10.60	83.30	77.50	61.70	65.80	65.00	36.60	23.20	7.90	6.30	5.60	30.03	27.53	26.63	26.63	27.53	35.70
11.60	8.60	72.50	73.30	69.20	77.50	95.80	104.80	28.80	4.90	7.10	8.30	7.53	10.83	12.53	13.33	13.33	69.60
13.10	7.50	82.50	75.00	63.30	62.50	72.50	95.50	20.60	11.90	8.00	5.90	21.63	21.63	26.63	27.53	23.33	102.10
6.80	4.80	67.50	71.70	61.70	66.70	60.00	29.50	13.80	10.10	4.70	1.90	12.53	12.53	11.63	12.53	8.33	23.30

P2AW2	P2AW3	P2AW4	P2AW5	N1TB1	N1TB2	N1TB3	N1TB4	N1TB5	N1AB1	N1AB2	N1AB3	N1AB4	N1AB5	P1TB1	P1TB2	P1TB3	P1TB4	P1TB5
17.90	12.20	4.50	2.50	26.70	26.70	28.30	19.20	20.00	40.00	15.90	5.10	4.70	3.60	46.70	47.50	40.00	44.20	42.50
7.70	1.60	1.10	2.00	18.30	19.20	20.80	19.20	19.20	25.50	12.90	10.70	5.70	4.10	48.30	45.00	44.20	39.20	48.30
32.70	12.00	6.80	5.60	10.80	10.80	20.80	29.20	22.50	34.60	21.20	17.20	11.90	7.20	46.70	46.70	41.70	43.30	35.80
16.40	2.90	4.30	3.90	22.50	25.00	20.80	19.20	18.30	36.40	20.00	12.50	9.00	8.70	42.50	48.30	38.30	45.00	45.80
3.80	2.40	3.30	1.90	24.20	19.20	20.00	19.20	19.20	22.50	6.50	8.80	6.10	5.60	48.30	47.50	37.50	38.30	40.00
12.10	2.70	1.40	2.90	20.80	20.80	19.20	20.00	16.70	46.10	22.00	11.60	4.20	4.00	50.00	49.20	40.00	38.30	45.00
14.90	4.10	3.30	1.70	29.20	26.70	27.50	22.50	15.80	37.80	14.50	8.80	3.60	2.20	50.00	50.00	49.20	40.80	41.70
9.80	5.60	4.90	2.60	18.30	27.50	20.00	17.50	17.50	31.70	13.60	6.70	3.70	3.20	56.70	41.70	39.20	39.20	44.20
6.90	7.70	3.10	1.80	18.30	19.20	19.20	20.00	20.00	29.20	13.50	7.00	5.10	3.10	60.80	41.70	42.50	42.50	52.50
12.70	5.80	3.30	2.50	20.00	19.20	26.70	16.70	22.50	49.80	17.50	8.20	3.80	4.20	40.00	40.00	39.20	37.50	37.50
19.60	7.60	4.90	4.90	22.50	21.70	20.80	20.00	19.20	65.60	33.10	12.00	4.20	7.00	44.20	42.50	37.50	38.30	39.20
17.00	14.40	4.50	3.60	13.30	18.30	18.30	15.80	25.80	41.50	20.00	7.40	7.10	11.00	41.70	41.70	48.30	43.30	42.50
9.80	4.90	2.60	3.00	23.30	23.30	28.30	20.00	18.30	40.20	23.20	10.00	7.00	6.30	48.30	43.30	45.00	43.30	44.20
14.60	6.00	4.40	5.80	16.70	16.70	17.50	20.80	20.80	42.00	13.20	9.00	6.60	5.90	51.70	51.70	45.00	45.00	44.20
18.60	6.70	6.10	4.50	27.50	30.00	27.50	19.20	17.50	36.80	25.60	8.50	4.80	5.80	55.00	51.70	50.00	39.20	43.30
8.30	4.20	0.90	1.60	17.50	25.80	25.80	25.00	14.20	37.10	13.60	13.70	7.20	6.00	50.80	45.00	45.00	43.30	43.30
16.70	5.80	4.20	2.80	19.20	24.20	20.80	28.30	28.30	24.90	9.30	3.90	4.30	3.40	61.70	57.50	56.70	50.00	50.00
10.40	2.80	0.40	0.60	18.30	24.20	24.20	23.30	15.00	34.70	15.20	7.20	6.30	4.60	59.20	59.20	54.20	57.50	55.80
13.70	3.80	4.70	1.40	25.80	25.80	36.70	27.50	27.50	51.90	16.50	10.60	9.00	5.70	44.20	50.80	50.80	56.70	45.80
8.50	3.70	0.90	0.30	21.70	17.50	19.20	19.20	20.80	21.80	10.80	7.90	3.50	2.60	51.70	40.00	40.80	40.00	40.00
19.20	7.30	2.30	3.90	20.00	23.30	22.50	21.70	21.70	37.60	12.70	4.50	5.10	5.70	43.30	46.70	42.50	44.20	45.80
6.70	6.00	0.80	2.10	30.00	30.00	21.70	15.80	21.70	22.80	12.60	6.40	2.10	2.40	48.30	47.50	42.50	40.80	41.70
13.10	5.20	1.60	2.50	25.80	25.00	26.70	26.70	18.30	18.20	11.60	5.60	6.80	3.40	52.50	52.50	47.50	47.50	49.20
6.60	6.30	6.50	4.00	31.70	21.70	22.50	21.70	21.70	35.30	17.50	7.80	4.90	4.80	55.00	46.70	45.00	39.20	40.80
9.30	6.30	4.90	3.20	20.80	20.80	20.80	20.80	16.70	70.60	29.00	13.10	10.20	4.60	46.70	48.30	38.30	37.50	35.80
38.00	13.20	9.10	5.00	14.20	20.00	20.80	29.20	20.00	64.50	24.40	15.40	10.20	5.70	52.50	49.20	49.20	42.50	40.80
24.20	14.10	5.10	3.80	24.20	24.20	14.20	15.00	15.00	54.10	28.00	15.90	13.30	9.30	48.30	48.30	42.50	44.20	43.30
16.50	9.30	7.20	5.30	30.00	29.20	21.70	21.70	21.70	76.50	24.00	12.20	11.30	7.40	49.20	44.20	44.20	37.50	50.80
10.40	4.40	4.20	2.00	20.80	20.80	21.70	21.70	20.80	11.10	3.80	5.50	4.40	3.10	50.00	48.30	45.00	44.20	44.20
11.50	6.90	4.50	1.40	28.30	29.20	29.20	28.30	21.70	37.00	14.40	7.70	3.30	3.50	50.80	50.00	41.70	41.70	41.70
11.10	4.20	2.30	1.70	19.20	19.20	16.70	23.30	27.50	23.70	6.10	6.20	2.60	1.30	50.00	51.70	38.30	45.80	50.00
5.30	2.70	2.40	1.80	16.70	20.00	20.00	27.50	28.30	54.90	31.90	18.50	8.50	7.50	46.70	47.50	44.20	41.70	46.70
19.70	8.80	4.70	3.30	26.70	27.50	16.70	17.50	18.30	50.40	25.60	13.70	9.10	5.80	49.20	42.50	40.00	40.80	45.80
17.70	5.10	3.60	3.00	21.70	21.70	20.80	21.70	21.70	30.50	16.00	10.80	7.70	4.20	45.00	45.80	39.20	40.80	40.80
11.40	3.20	1.80	0.70	20.00	22.50	21.70	21.70	21.70	20.00	12.50	8.10	4.00	2.20	48.30	40.80	40.00	39.20	40.00

6.00	3.10	-0.40	1.40	25.00	15.80	21.70	20.80	20.00	26.40	8.30	6.40	5.30	4.00	42.50	45.80	46.70	42.50	44.20
12.20	6.00	5.00	4.90	30.80	24.20	24.20	23.30	15.00	50.60	16.10	8.90	8.60	5.70	50.80	48.30	47.50	44.20	45.00
15.20	12.90	4.60	4.30	19.20	15.00	23.30	23.30	24.20	34.30	14.80	19.30	9.90	8.60	53.30	48.30	41.70	42.50	45.00
9.00	9.80	7.20	4.50	21.70	20.00	16.70	16.70	15.80	105.00	33.80	35.50	10.40	7.70	45.80	39.20	39.20	28.30	42.50
29.20	7.40	1.80	3.00	24.20	18.30	18.30	17.50	16.70	75.60	33.40	10.80	8.00	10.40	56.70	41.70	42.50	38.30	39.20
7.70	8.00	4.10	2.90	18.30	25.00	24.20	20.80	20.80	25.70	8.30	6.00	6.00	4.40	52.50	50.00	39.20	40.80	42.50

P1AB1	P1AB2	P1AB3	P1AB4	P1AB5	N2TB1	N2TB2	N2TB3	N2TB4	N2TB5	N2AB1	N2AB2	N2AB3	N2AB4	N2AB5	N2TB1	N2TB2	N2TB3	N2TB4	N2TB5
54.30	26.30	15.00	12.90	9.40	77.50	74.20	75.80	103.30	102.50	46.00	17.50	4.20	3.00	4.50	24.13	21.63	20.83	25.03	25.03
71.30	36.70	19.50	12.30	8.20	75.80	75.00	86.70	95.80	95.00	41.90	13.90	9.50	6.00	6.00	48.33	47.53	46.63	46.63	45.83
76.80	32.90	22.00	16.00	8.30	90.80	97.50	60.80	61.70	52.50	49.30	31.00	19.00	12.30	7.60	13.33	12.53	15.03	15.03	15.03
77.20	44.70	21.70	17.50	14.20	85.00	80.80	84.20	84.20	78.30	53.90	22.50	11.50	10.30	7.30	25.03	25.03	21.63	26.63	28.33
62.20	24.60	18.20	11.90	8.00	67.50	66.70	70.80	72.50	72.50	55.00	12.80	6.40	4.10	5.30	24.13	25.03	17.53	19.13	22.53
79.50	36.90	21.90	11.40	8.80	75.00	83.30	85.00	81.70	91.70	38.50	19.30	10.10	6.20	4.70	22.53	23.33	24.13	30.03	29.13
86.50	39.80	20.50	12.40	12.10	78.30	67.50	67.50	96.70	97.50	10.20	7.60	4.50	2.50	1.40	26.63	23.33	22.53	23.33	24.13
59.00	26.50	15.00	10.40	7.80	73.30	72.50	65.80	66.70	64.20	82.60	37.00	11.40	2.10	1.80	35.03	33.33	39.13	32.53	30.03
44.50	29.70	21.00	13.30	8.90	79.20	99.20	98.30	105.00	101.70	47.20	23.40	9.30	7.70	5.90	31.63	31.63	35.03	35.83	36.63
93.50	45.30	22.90	12.90	9.50	79.20	65.80	70.80	95.80	96.70	74.20	29.30	6.80	11.10	9.00	30.03	29.13	28.33	28.33	26.63
87.70	45.20	16.80	13.40	12.50	72.50	63.30	63.30	74.20	82.50	60.70	36.40	16.80	7.30	8.60	7.53	16.63	16.63	16.63	15.03
59.40	44.50	25.70	10.40	8.80	70.00	63.30	67.50	68.30	76.70	71.80	10.90	-5.60	6.10	8.90	24.13	23.33	29.13	27.53	24.13
55.60	29.40	19.20	14.00	11.40	73.30	74.20	60.80	87.50	87.50	61.40	32.80	11.60	7.70	4.80	18.33	17.53	18.33	17.53	18.33
74.20	28.20	17.40	12.30	8.40	71.70	70.80	66.70	67.50	68.30	43.10	23.50	10.60	5.60	4.80	26.63	27.53	27.53	26.63	26.63
59.60	30.30	15.40	10.90	8.60	93.30	93.30	60.80	59.20	84.20	124.10	48.10	6.60	7.80	6.10	22.53	22.53	21.63	22.53	22.53
84.20	38.60	19.70	12.70	9.20	78.30	82.50	67.50	83.30	95.80	45.30	20.80	10.50	5.60	6.00	24.13	24.13	24.13	17.53	17.53
32.00	19.30	14.10	10.30	9.40	96.70	97.50	98.30	96.70	101.70	30.90	12.90	7.00	7.20	5.00	25.03	24.13	24.13	29.13	29.13
42.50	33.10	14.10	14.30	10.70	75.80	76.70	85.80	88.30	90.80	50.10	14.70	8.70	4.60	3.60	33.33	33.33	35.03	35.03	34.13
48.60	23.20	7.90	7.10	3.40	65.80	94.20	105.80	95.80	70.80	74.60	24.60	22.00	14.10	6.30	28.33	27.53	36.63	29.13	29.13
54.70	30.80	18.40	10.80	8.00	68.30	68.30	68.30	85.80	90.00	36.90	20.70	10.60	4.30	4.40	28.33	29.13	27.53	27.53	25.83
52.10	26.30	11.50	9.80	9.60	85.80	85.80	67.50	81.70	80.00	75.70	21.60	9.90	5.20	5.00	25.83	30.83	29.13	20.83	15.03
55.00	29.30	19.70	10.80	9.10	77.50	83.30	84.20	99.20	95.80	59.00	24.40	9.50	6.90	3.70	29.13	29.13	30.03	30.83	22.53
66.80	32.60	16.20	11.80	8.60	89.20	79.20	69.20	68.30	69.20	48.30	20.90	15.60	9.20	3.20	23.33	23.33	23.33	22.53	18.33
41.10	26.80	17.20	11.60	9.50	75.80	76.70	65.80	95.80	89.20	89.30	34.20	11.60	7.00	7.20	19.13	18.33	20.83	17.53	19.13
77.00	44.50	21.50	15.30	10.70	75.00	66.70	68.30	55.80	55.80	88.70	23.80	10.10	10.20	6.00	17.53	13.33	19.13	19.13	18.33
76.60	47.10	27.00	16.40	11.50	78.30	70.00	70.00	70.80	85.80	74.00	33.70	19.40	9.40	6.90	31.63	31.63	29.13	29.13	27.53
92.40	47.60	28.10	17.40	13.00	76.70	76.70	72.50	65.80	64.20	98.50	47.30	20.10	15.30	14.80	25.03	25.03	25.03	21.63	20.83
140.70	57.70	23.30	18.10	16.70	77.50	65.80	65.80	72.50	70.00	90.80	36.20	11.90	6.40	5.90	16.63	15.03	22.53	10.83	10.83
65.30	27.10	16.80	9.60	6.80	95.80	82.50	94.20	80.00	70.80	20.60	10.10	8.70	6.40	2.90	19.13	20.03	23.33	19.13	20.03
77.30	28.40	17.00	10.10	7.70	92.50	77.50	66.70	67.50	94.20	45.60	22.60	5.20	1.80	5.90	29.13	29.13	32.53	32.53	30.83
23.50	8.30	5.80	3.70	3.90	70.00	70.00	69.20	80.00	94.20	26.20	12.20	9.30	3.90	4.90	28.33	29.13	30.03	30.83	30.83
58.10	33.20	22.00	12.10	10.10	74.20	74.20	70.00	85.80	96.70	90.20	39.10	16.40	9.90	10.60	21.63	20.83	24.13	25.03	22.53
116.20	49.10	25.00	18.00	11.10	81.70	81.70	57.50	56.70	68.30	74.30	32.60	10.10	9.30	9.90	27.53	27.53	28.33	23.33	26.63
58.30	33.10	20.20	12.70	8.90	79.20	70.80	75.00	67.50	95.80	74.20	25.70	13.50	6.40	4.20	20.03	22.53	19.13	18.33	16.63
70.10	37.80	19.00	11.20	7.10	80.80	69.20	81.70	99.20	104.20	47.50	21.50	12.00	7.80	7.00	15.03	15.03	24.13	21.63	25.03

21.10	15.70	11.90	7.60	6.00	74.20	68.30	68.30	67.50	80.80	61.40	18.70	7.60	4.50	3.40	22.53	22.53	20.83	17.53	18.33
81.50	32.10	20.10	15.10	11.20	98.30	71.70	70.00	78.30	81.70	64.60	19.50	9.90	6.20	8.20	51.63	43.33	42.53	42.53	41.63
103.10	42.10	25.00	15.90	15.90	81.70	85.00	65.00	68.30	57.50	14.00	13.30	23.90	9.30	6.50	17.53	16.63	10.83	17.53	19.13
121.50	74.60	54.70	24.50	12.90	61.70	66.70	80.80	89.20	81.70	107.00	16.60	36.60	14.40	7.70	15.03	12.53	19.13	17.53	17.53
135.30	36.00	23.10	15.40	16.30	90.80	85.00	85.00	85.80	77.50	76.90	46.20	17.60	8.70	10.80	35.83	35.03	35.03	40.03	25.03
58.00	25.90	12.90	11.40	7.50	87.50	81.70	81.70	63.30	63.30	42.30	13.30	8.90	3.90	3.10	36.63	35.83	35.83	32.53	29.13

N2AB1	N2AB2	N2AB3	N2AB4	N2AB5
20.80	6.80	3.60	2.60	2.90
38.90	17.50	12.50	5.40	4.60
49.50	24.60	10.20	7.20	6.80
57.20	21.40	12.30	8.00	9.50
32.40	14.80	7.90	3.10	3.90
19.20	16.50	7.00	6.10	5.70
41.20	16.20	6.00	5.90	7.00
77.10	15.90	4.30	3.60	4.60
20.40	18.00	9.40	5.10	3.30
48.30	18.60	8.70	8.30	3.80
57.20	19.70	12.80	6.50	5.80
75.80	31.90	17.20	8.50	10.20
59.10	19.30	7.90	3.90	3.80
25.40	11.70	5.90	8.20	6.80
68.50	21.60	7.30	8.50	9.10
41.40	17.10	7.10	3.30	2.80
20.70	8.60	6.20	4.20	2.10
30.40	19.50	7.00	2.70	2.20
23.70	22.30	11.10	4.10	4.10
32.60	11.90	8.50	5.60	6.30
40.50	9.70	10.30	5.60	4.60
35.90	13.00	7.50	5.10	3.00
32.80	12.90	9.10	3.40	5.00
34.30	17.40	6.10	3.40	4.40
24.40	17.10	11.70	5.30	6.70
43.20	10.80	9.50	8.40	6.60
77.10	24.10	14.90	8.30	2.90
83.80	29.20	12.90	11.10	8.30
23.80	6.10	5.10	3.30	4.20
41.50	18.50	6.50	5.90	6.90
40.30	15.00	5.20	1.00	0.60
68.50	25.00	8.20	7.20	1.50
61.40	16.40	7.80	7.40	6.10
36.90	11.20	5.70	1.80	3.70
31.80	13.70	7.70	5.90	3.60

32.20	10.10	5.80	3.60	3.60
33.60	28.30	14.90	11.90	8.20
49.40	10.50	12.70	4.80	7.90
102.70	25.10	14.50	11.40	7.10
75.60	41.30	13.70	9.00	5.90
23.60	13.50	5.10	2.00	1.60
